# On Perforation and Division of Permanent Stricture of the Urethra by the Lancetted Stilettes; with Observations on the Nature and Treatment of Spasmodic and Inflammatory Stricture, and in Various Other Urethral Affections

**Published:** 1836-04

**Authors:** 


Art. XV.—*On Perforation and Division of Permanent Stricture of the Urethra by the Lancetted Stilettes: with Observations on the Nature and Treatment of Spasmodic and Inflammatory Stricture, and in various other Urethral Affections.* By R. A. Stafford, Surgeon to the St. Marylebone Infirmary, &c. &c. *Third Edition.*—London. Longman, 1836. 8vo. pp. 324.

Our surgical brethren are much indebted to Mr. Stafford for the industry and ability with which he has investigated the mode of treating permanent strictures of the urethra by the lancetted stilettes. In this third edition of his work, he gives additional proofs of its efficacy and safety, and of the rapidity and permanency of the cure effected by it. We have seen this practice adopted in several cases, and are quite prepared to admit that it is not only applicable, but very useful, in certain cases of permanent stricture where the life of the patient is in danger, and where no other means can be employed: but we cannot admit, with Mr. S., that it is only "an *apparently* difficult mode of treatment." Practice may have, and no doubt has, given to him great dexterity in the use of the lancetted stilettes; but, in the hands of surgeons in general the safe use of the instrument would be by no means so certain. We remember to have heard the much lamented Dupuytren observe, in speaking of the lancetted stilettes in the treatment of permanent stricture of the urethra, that the instrument would be perfectly safe "s'il avait des yeux!" and we confess we have felt this irremediable deficiency when we have seen it employed. In Mr. Stafford's book every necessary instruction will be found both as to the distinction of the cases in which his treatment is applicable and as to the manner of carrying it into effect.

